# Out

**DOI:** 10.1007/s10912-025-09963-4

**Published:** 2025-07-09

**Authors:** Filippo Naitana

**Affiliations:** Quinnipiac University, 275 Mt Carmel Ave, Hamden, CT 06518 USA

I have been meaning

to write you for a long time.

But you know how life

gets out here. No big-eyed dogs

gently chewing on the void.

No anchoring wish

for freedom. You awake and

the gray cathedral

of talking branches has grown

louder. The poets are gone.

## Data Availability

No datasets were generated or analysed during the current study.

